# Clinical Usefulness of a Smartphone-Based 6-Minute Walk Test in a Hospital Outpatient Clinic Within the Constraints of the COVID-19 Pandemic: Mixed Methods Study

**DOI:** 10.2196/70495

**Published:** 2025-10-10

**Authors:** Dario Salvi, Carl Magnus Olsson, Jackson Molloy, Elizabeth Orchard

**Affiliations:** 1 Department of Computer Science and Media Technology, Sustainable Digitalisation Research Centre, Malmö University, Nordenskiöldsgatan 1, Malmo, 205 06, Sweden, 46 040 665 70 00; 2 Cardiac Physiology, Oxford University Hospitals NHS Trust, Oxford, United Kingdom; 3 Department of Cardiology, Oxford University Hospitals NHS Trust, Oxford, United Kingdom

**Keywords:** Timed Walk app, 6-minute walk test, 6MWT, physical capacity, mobile health, technology acceptance, usability, mixed methods

## Abstract

**Background:**

The 6-minute walk test (6MWT) measures exercise capacity in cardiorespiratory, neurological, and musculoskeletal conditions. It consists of observing how far a patient can walk in 6 minutes and is usually performed in a corridor in a clinic. During the COVID-19 pandemic, as health care systems cancelled nonurgent outpatient appointments, many tests were conducted online. At Oxford University Hospitals National Health Service Foundation Trust, patients followed up on by cardiovascular outpatient clinics were asked to use the open-source Timed Walk app to perform the 6MWT in their community as a substitute for the regular tests in the clinic.

**Objective:**

This study aimed to assess the clinical usefulness of the app within the context of the pandemic.

**Methods:**

Consented patients were invited to perform a 6MWT outdoors using the app at least once a month and report the results through periodic telephone calls and visits. Clinical decisions made for the same cohort were registered, with a focus on the effect of the app in supporting decision-making. Data collected through the app during the study period were compared with 6MWTs performed in the prepandemic period.

**Results:**

This study was conducted between October 2021 and December 2022. A total of 55 participants consented (n=25, 45% female; mean age 44.80, SD 17.49 y). In total, 741 events were logged. A total of 51 medical decisions were made for 25 patients; in 41% (21/51) of the decisions, the app played a role, affecting 44% (11/25) of the patients. Between 2018 and 2022, a cohort of 49 patients for whom data were available performed 63 6MWTs in the clinic (18 in 2021), whereas the same patients performed 605 tests using the app in 2022 (ie, October 2021 to December 2022).

**Conclusions:**

The use of the Timed Walk app for remote 6MWTs allowed clinicians to obtain frequent and objective indications of the status of the patients during the pandemic, compensating for the absence of regular clinic appointments and providing 33 times more tests than in the prepandemic period. These tests supported approximately half of the clinical decisions made regarding the consented patients, showing that the app is useful in clinical practice.

## Introduction

### Background

The switch to home-based, remote follow-up during the COVID-19 pandemic quickly became crucial as outpatient appointments were cancelled to minimize exposure to infected persons [1] and reduce the burden on health care providers. This was especially important for vulnerable and high-risk patients and showed encouraging clinical outcomes [1-3]. In the context of respiratory and cardiac diseases, it was suggested that exercise tests could be delivered remotely in home settings instead of clinics [4,5]. Simple tests such as the 6-minute walk test (6MWT), the sit-to-stand test, the Timed Up and Go test, and the step test were particularly useful, given their reliability and strong correlation with general symptoms and the possibility of patients performing them independently owing to the use of technology such as accelerometers and smartphone apps.

The 6MWT in particular is a standard method for measuring exercise capacity in conditions such as pulmonary arterial hypertension, heart failure, and cardiorespiratory and musculoskeletal conditions. The test consists of measuring how far a patient can walk in 6 minutes. The measured 6-minute walk distance (6MWD) is indicative of the functional status of the patient and can be used for follow-up and for measuring response to rehabilitation and pharmacological interventions [6-8].

The test can be delivered through smartphone apps, as shown in a previous research project where a mobile phone app was designed, developed, and evaluated to allow patients with pulmonary hypertension to perform the 6MWT in the community [9,10]. This is particularly useful for patients who cannot attend regular visits at the clinic, as was the case during the COVID-19 pandemic, or to reduce costs associated with undertaking the test itself. Remote testing reduces the burden of traveling to clinics for patients and eliminates the need for health care professionals to receive the patients, guide the tests, and process the results [9]. Remote 6MWTs are, in fact, recommended in recent guidelines for cardiac and pulmonary rehabilitation [11,12].

Notwithstanding its promises, telemedicine still faces significant barriers, such as security and privacy concerns, lack of proven effectiveness, and poor design and fit into clinical workflows [13,14]. Understanding benefits of telemedicine as complementary to in-clinic visits for both clinicians and patients may be a reasonable postpandemic perspective for research. To address this need, our study evaluated the adoption of a remote mobile-based 6MWT among hospital outpatients from a clinical and patient perspective during the COVID-19 pandemic.

### Objectives

This study had three specific aims: (1) to assess the clinical usefulness of a smartphone app for remote 6MWT in the context of the pandemic, (2) to assess user acceptance of the app, and (3) to validate and improve the algorithms that compute the walked distance from the sensor data collected by the phone. This paper, which is part of a series of 3 manuscripts, focuses on the first aim, concretely, the *clinical usefulness* of the app.

## Methods

### Overview

The goal of this study was to contribute to existing research on the adoption of remote mobile-based 6MWTs through a mixed methods approach where we assessed complementary quantitative and qualitative data to validate the use of an app in the context of the pandemic. As this first paper focuses on clinical usefulness, we report on the study design particularly focusing on this aim.

The app used in this study relies on the outdoor 6MWT algorithm developed in the studies by Salvi et al [9,10]. This was motivated not only by the satisfactory results shown in previous research but also by the important facts that the algorithm is published under an open license and is simple to implement. For this study, we reimplemented the same algorithm in a new smartphone app described in the following section.

### The Timed Walk App

The Timed Walk app is available free of charge for both Android and iOS, and its source code is distributed with an open-source Massachusetts Institute of Technology license [15]. The app has 1 main functionality: to measure the distance walked in 6 minutes using the global navigation satellite system (GNSS) of the phone. The data collected by the app are stored locally on the user’s phone. Users decide whether they want to share either the history of all past tests (containing date, distance, and duration) or the details of the last performed test (containing data from sensors including localization coordinates, speed, and acceleration) using a button that opens the standard dialogue of the operating system where the channel of communication (eg, email or SMS text message) can be selected.

The walked distance is computed using the information provided by the GNSS, which thus requires that the test be performed outdoors, where satellites are within reach. As GNSS data can be affected by noise, the app uses an algorithm to filter out unreliable estimated positions and computes a total walked distance as described and validated in the studies by Salvi et al [9,10] (under a different app name).

**Figure 1.**
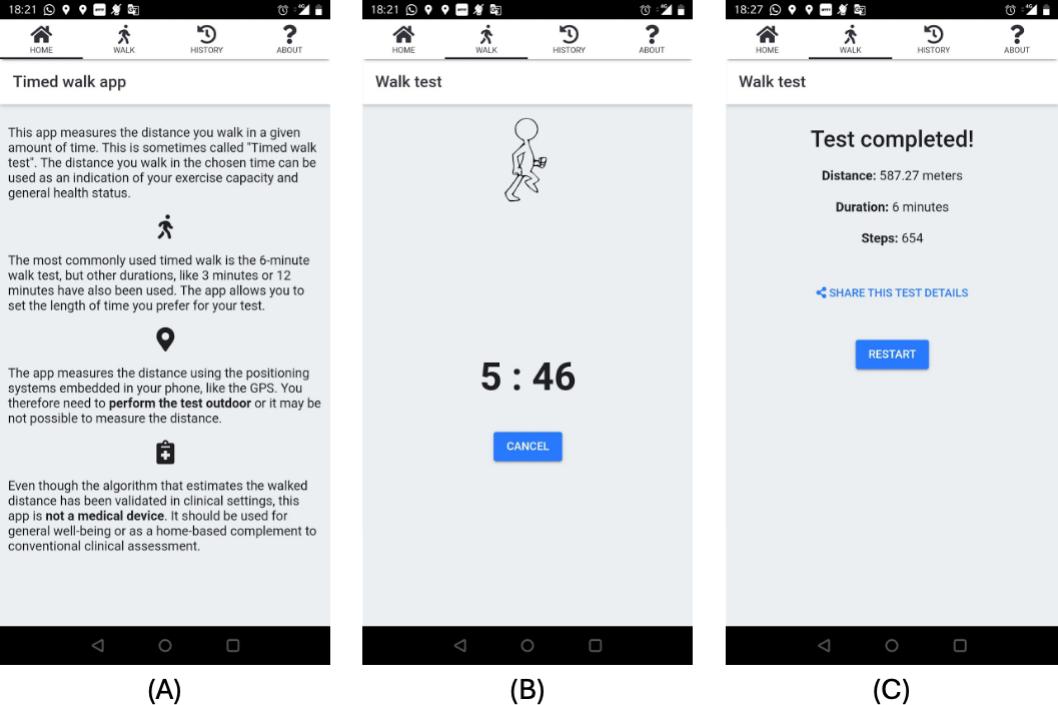
Screenshots of the Timed Walk app showing the instructions for the test (A), the running test with a countdown timer (B), and the information after completion of the test (C).

The app includes extensive instructions about its purpose and how to perform the test, with both text and voice instructions during the test following the American Thoracic Society guidelines [16]. Even if the test is considered generally safe, patients are instructed to stop walking if they do not feel well and to seek medical help. Screenshots of the app are shown in Figure 1.

### Clinical Usefulness

While the accuracy, reliability, and usability of apps for the 6MWT have been examined in research [9,10,17-19], little is known about the usefulness of app-based measurements in a clinical context. The usefulness of the 6MWT is commonly assessed retrospectively by investigating the relationship between walked distance and clinical variables, including hospitalizations, quality of life, or response to therapy [20]. While the improvement in these variables is an undoubtedly desirable goal, they are measurable only in large-scale, longitudinal studies where confounders can be mitigated. In the context of smartphone apps, frameworks and standards for evaluation exist, but they rarely describe which quantities or indicators to measure as these can vary greatly and are context dependent [21].

In our context, a simple and effective way to measure the clinical usefulness of an app is to measure *the effect of the app in supporting clinical decision-making* [22]. This is particularly suitable for the scenario in which a 6MWT app would provide frequent and objective measurements about patients, information that is “clinically actionable” [23] (ie, that is considered as a relevant input while making clinical decisions).

Measuring usefulness according to this definition (as support for clinical decision-making) can be done by recording clinical events such as visits, evaluations, and treatment changes, which allows for the examination of how clinical decisions are affected by the presence of certain information or workflows [24,25]. To the best of our knowledge, logging of clinical decisions has never been used to evaluate the clinical usefulness of apps despite providing the opportunity to measure the impact of adopting an app in an objective way within a specific context of use, such as the COVID-19 pandemic.

### Study Design

Data were collected from patients attending the cardiology outpatient department at Oxford University Hospitals National Health Service Foundation Trust (OUH), Oxford, United Kingdom, during the restrictions put in place due to the COVID-19 pandemic. Patients were either under regular follow-up for their cardiac disease or had been referred to see clinicians in the outpatient setting for assessment and treatment.

Outpatients who were undergoing follow-up in the cardiovascular outpatient clinics (either face-to-face or remotely) in Oxford and were able to walk and use a smartphone were considered eligible. Some of these patients were already using the app before the start of the study, for example, in the case of the pulmonary hypertension clinic.

The inclusion criteria were as follows: (1) being enrolled in an OUH cardiovascular outpatient clinic, (2) owning or having access to a smartphone with either Android or iOS, (3) being able to use a smartphone app, and (4) being able to walk.

The exclusion criteria were as follows: (1) long-term oxygen therapy, (2) cognitive impairments, (3) inability to use a smartphone, (4) pregnancy, and (5) inability to complete a 6MWT.

We did not sample participants as the aim was to involve all patients in the cardiovascular clinics who required a 6MWT and were able to use the app. However, as restrictions started to be lifted in 2022 and practical reasons made the recruitment pace slower than desired (approximately 4 patients per month), a decision was made to stop recruitment after the 100th patient was selected.

Patients were screened by reviewing outpatient clinic lists for possible inclusion every week based on already scheduled clinic visits (either face-to-face or virtual). A list of candidates who met the inclusion criteria was prepared before their scheduled outpatient clinic. Prospective participants were approached by a member of the clinical team for consideration and recruited during their regular outpatient consultation either via phone call or during a visit to the clinic.

During the consultations, patients were asked about their access to and comfort with smartphones to verify compliance with the inclusion criteria. Those who were confirmed to comply with all inclusion criteria were invited to participate, signing a consent form as they were enrolled. Consent forms were delivered via post.

Participants in the study were asked to download the Timed Walk app and use it to perform a 6MWT in the community outdoors at least once a month and report the measured 6MWD to the clinicians during the outpatient consultations via phone or at the clinic.

Patients enrolled at the clinic were introduced to the app during the outpatient appointment and shown how to use it. This was different from the usual clinical care, as they would normally simply undertake a 6MWT. Patients enrolled via phone were instructed verbally. As the app is quite minimalistic, only minor recommendations about performing the test outdoors were needed.

To assess the usefulness of the app for the clinical management of consented patients, at each consultation (either through phone or face-to-face visit), the clinician who contacted the patient logged any study-related event on a spreadsheet marked with a code. Coding was used to simplify and automate later analysis and was defined in collaboration with the clinical professionals that were part of the study.

The spreadsheet contained the following columns:

- Patient ID: study identifier of the patient
- Date: date of the event
- Event type: using a custom code schema, these events were related to recruitment, withdrawal, home or clinic 6MWT, app use, technical problems, clinical investigations, multidisciplinary team discussions, and changes in treatments
- Comments: any additional comments (free text)
- Distance: if the event was related to a 6MWT, the walked distance in meters was reported in this column

In addition, the following demographic information was recorded for each patient: age, sex, main conditions, and study participation (declined participation, consented, completed the study, or withdrew).

Other aspects included in this study but not part of this paper were usability and acceptance questionnaires, interviews, and data quality analysis.

This study sits between the *appropriateness* and *feasibility* levels of the taxonomy of implementation science outcomes [26], where constructs such as perceived fit, relevance, usefulness, actual fit or utility, suitability for everyday use, and practicability are measured at an individual health care provider such as a clinic or organization. As suggested by Proctor et al [26], common measurement tools at this stage are surveys, interviews, and administrative data, which is compatible with our study design.

### Data Analysis

For the purpose of assessing the clinical usefulness of the app within the context of the pandemic, we identified 2 objectives.

#### Objective 1: Assess Whether Differences in Distance Walked According to the App Altered the Management of Patients

This was estimated by counting the number of clinical investigations, multidisciplinary team discussions, and changes in treatments triggered by variations in app-based 6MWD. The spreadsheet where events were logged was used for this purpose, including data from all the patients who consented. We computed descriptive statistics, including the number of clinical decisions; the number of patients involved in those decisions; and, given that patients were involved in the study over a varying number of days, the number of decisions per patient and year.

#### Objective 2: Compare the Use of the 6MWT During the Pandemic With Prepandemic Use

This was done by comparing the total number of 6MWTs performed during the pandemic period compared with the prepandemic period. The number of in-clinic 6MWTs was retrieved from the electronic health record of the hospital, whereas the number of app-based 6MWTs was extracted from the event log. We considered only those patients who consented and whose data were available on the clinical health record over the entire period under analysis, comprising 2018 to 2022.

### Ethical Considerations

This study was a collaboration between the OUH, United Kingdom, and Malmö University (MAU), Sweden. MAU is the main developer and maintainer of the Timed Walk app and provided engineering support in terms of both the app and data analysis, whereas OUH recruited patients and collected data. This study was conducted in accordance with the Declaration of Helsinki, with ethics approval from the UK National Health Service Health Research Authority (protocol reference 17/WM/0355). Written informed consent was obtained from all participants involved in the study, and no compensation was provided to them.

All data were sent by patients to the care team at the OUH through email or verbally shared via phone calls; data were subsequently pseudonymized with study participant IDs and analyzed by staff at MAU. After analysis, data were archived following UK and Swedish regulations.

## Results

### Demographics

This study was conducted between October 2021 and December 2022. A total of 100 patients initially agreed to participate. Of these 100 patients, 88 (88%) were contacted over the phone, and 12 (12%) were unreachable. Of the 88 patients contacted, 77 (88%) confirmed their agreement to participate and received printed study information and informed consent forms via post. Of these 77 patients, 55 (71%) signed and returned the consent forms, and their data were used for analysis for objective 1. Of the 55 participants, 3 (5%) withdrew during the study but agreed to have their data used for analysis. Of the 55 consented patients, 49 (89%) had clinical health records at the hospital and were included for objective 2.

**Figure 2.**
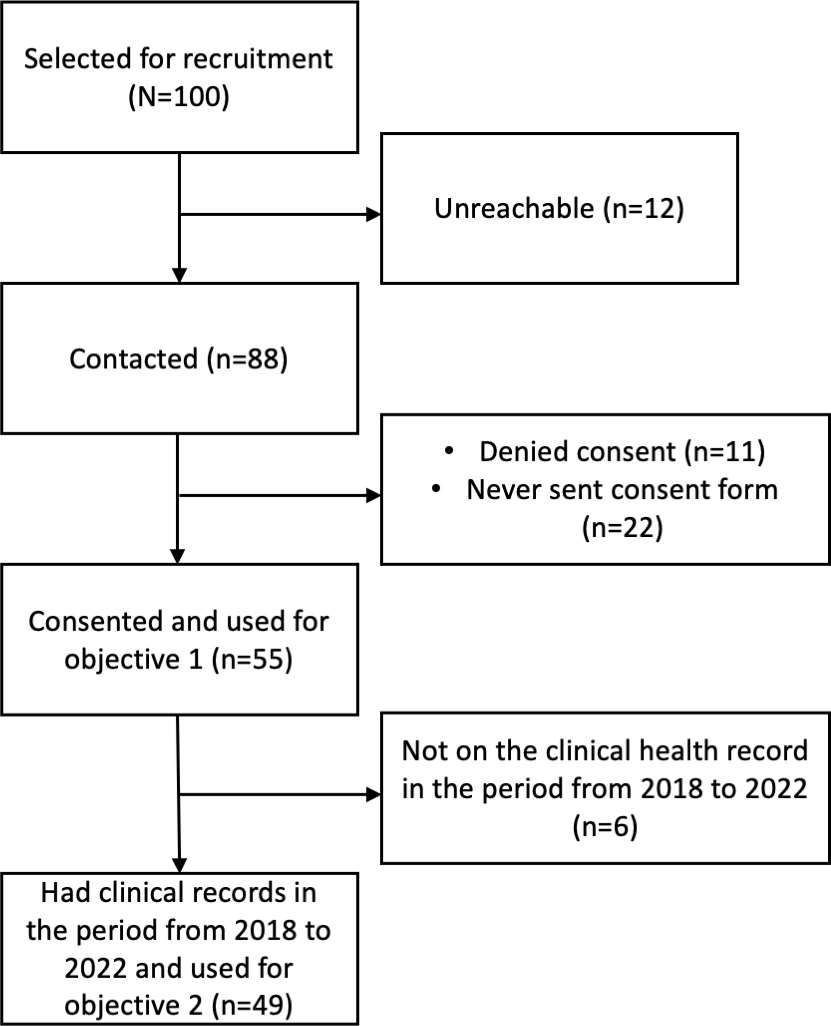
CONSORT (Consolidated Standards of Reporting Trials)–like diagram of the study.

A CONSORT (Consolidated Standards of Reporting Trials)–like diagram is shown in Figure 2.

Of the 55 consented participants, 25 (45%) were female. The average age was 44.80 (SD 17.49) years. Patients were involved in the study for a varying number of days (mean 314, SD 93; range 62-441 days). In total, 741 events were logged between September 29, 2021 (the earliest event), and December 30, 2022 (the latest event). In total, 612 mobile-based 6MWTs were completed, with an average of 11.26 (SD 15.11; range 0-84) mobile 6MWTs per patient. The average distance measured in those tests was 471.20 (SD 125.51; range 95-989) m. In total, 13% (7/55) of the patients did not perform any mobile 6MWT.

### Objective 1: Assess Whether Differences in Distance Walked According to the App Altered the Management of Patients

We used the data from the 55 patients who consented for the analysis, including the 3 (5%) patients who withdrew but were happy to have their data used.

From the logs, we computed descriptive statistics about the frequency of clinical decisions, including new evaluations and changes in treatment plans. The types of logged items are reported in Table 1 for each type. As patients were involved in the study over different time spans, we also report the average and SD of the number of events per year (365 days) per patient.

**Table 1.** Number of events, number of patients involved, and yearly average of times logged per event type per patient.

Logged item type	Number of events	Number of patients involved	Events per year per patient, mean (SD)
The patient agreed to join the study and provided consent.	54	54	1.31 (0.88)
The patient decided to withdraw from the study.	1	1	0.05 (0.34)
The patient declined consent and did not join the study.	0	0	0.0 (0.0)
The patient declared having the app installed on a phone they could use.	0	0	0.0 (0.0)
The patient declared that they had technical issues with the app.	0	0	0.0 (0.0)
The patient shared a 6MWT^a^ not older than 2 weeks through either email or telephone.	612	48	13.73 (14.36)
Regular clinic 6MWT.	14	10	0.29 (0.64)
The patient needed further evaluation because of changed medical conditions. App-based 6MWT was irrelevant to the decision.	7	4	1.1 (0.52)
The patient needed further evaluation because of changed medical conditions. App-based 6MWT was partially involved in the decision.	7	7	0.22 (0.64)
The patient needed further evaluation because of changed medical conditions. App-based 6MWT was fundamental to the decision.	4	3	0.15 (0.71)
The patient needed a change in treatment. App-based 6MWT was irrelevant to the decision.	23	15	0.53 (1.11)
The patient needed a change in treatment. App-based 6MWT was partially involved in the decision.	10	5	0.22 (0.75)
The patient needed a change in treatment. App-based 6MWT was fundamental to the decision.	0	0	0.0 (0.0)
Any clinical decision.	51	25	1.25 (1.79)
Any clinical decision for which app-based 6MWT was partially involved or fundamental.	21	11	0.59 (1.27)

a 6MWT: 6-minute walk test.

As shown in the table, most of the logged items (612/741, 82.6%) were app-based 6MWTs provided by 87% (48/55) of the patients. In comparison, only 1.9% (14/741) of clinic-based 6MWTs were logged in the same period for 18% (10/55) of the patients. A total of 6.9% (51/741) of the logs were related to clinical evaluations or changes in treatment, affecting 45% (25/55) of the patients. In 41% (21/51) of the decisions, affecting 44% (11/25) of the patients, the information provided by the app played a role. In 8% (4/51) of the decisions, affecting 12% (3/25) of the patients, the app played a fundamental role.

In terms of events per patient per year, we observed slightly more than one 6MWT per month (13.73, SD 14.36 per year), with some patients performing as many as 81 tests per year. Clinical decisions were less frequent, with a mean of 1.25 (SD 1.79) per year, approximately half of which involved the use of the 6MWT as measured through the app.

It is noteworthy that patient consent, withdrawal from study, app installation, use, and issues were not consistently recorded on the log but were also noted on a separate sheet.

### Objective 2: Compare the Use of the 6MWT During the Pandemic With Prepandemic Use

We provide statistics regarding the number of 6MWTs performed by our cohort of patients in the clinic over the last 5 years and compare it with the number of 6MWTs performed during the study period. This is to identify the difference between the number of 6MWTs per patient per year before the pandemic (standard 6MWT in the clinic) and after the pandemic (using the app).

The number of 6MWTs conducted in the clinic for that cohort was 17 in 2018, 16 in 2019, 8 in 2020, 18 in 2021, and 13 in 2022. The total number of 6MWTs performed using the app during the study period (October 2021 to December 2022) was 605 for the same group of 49 participants.

The reduction in in-clinic tests in the year 2020 was due to the restrictions imposed by the pandemic, which was later compensated for by more tests in 2021. Of note is that the number of app-based tests during the study period was 33 times larger than the highest number of in-clinic tests for the analyzed cohort (18 in 2021). This study also yielded 8 times more tests than the number of in-clinic tests conducted over 5 years.

## Discussion

### Principal Findings

The use of the app allowed for the retrieval of frequent information about patients remotely. In fact, while only 14 6MWTs were performed in the clinic on our 55 participants, 612 additional 6MWTs were performed using the app and communicated to the involved clinicians. The information sent played a role in the clinical decisions made regarding those patients. In 41% (21/51) of the clinical decisions, the distances provided by the app were considered a contributing element, and in 8% (4/51) of the cases, they were considered even fundamental to the decision. These decisions affected one-fifth of the patients who used the app (11/55, 20%) and approximately half (11/25, 44%) of the patients for whom clinical decisions were made during the study period.

The 6MWT has a recognized prognostic value. The walked distance has been shown to be a predictor of all-cause mortality and hospitalization [27] and to be responsive to clinical change [6]; therefore, it was expected that the information retrieved through the app would be used in clinical decisions. Given the circumstances during the pandemic, the app may have been the only means to obtain objective information about some of the patients involved. This clearly indicates its usefulness as a tool that can augment the information that clinicians obtain either as a complement to in-clinic 6MWTs or as a substitute, especially if supervised 6MWTs are not readily available, for example, in remote areas.

Data show that, for the cohort of 49 patients for whom we had historical data, the number of 6MWTs in the clinic varied between 8 and 18 per year, with the lowest number in 2020 due to the pandemic. During the study period, which roughly encompassed the entire year 2022, the number of 6MWTs in the clinic was 13, which means that 26% (13/49) of those patients were able to perform a test in the clinic. In comparison, 87% (48/55) of our patients performed at least one 6MWT on the app in the same period, and on average, our patients performed one 6MWT per month on the app. This proves not only that the app could compensate for the regular 6MWTs in the clinic during the pandemic but also that the amount of data that the app allows to be retrieved is 33 times larger than that for regular visits, with 605 app-based tests in 2022 compared to 18 retrieved in the clinic in 2021.

These results have to be considered in the context of the fact that the distribution of the number of tests per patient was wide, with 7 participants performing no tests at all and 2 patients performing one-quarter (147/612, 24%) of all tests (one performed 66 tests per year, and the other performed 81 tests per year).

### Limitations

We showed that 41% (21/51) of the clinical decisions made regarding our cohort during the study were at least partially based on the measurements provided by the app. However, this study did not use a control group; therefore, it is impossible to ascertain whether patients would have received the same level of care without the use of the app.

It is also important to observe that this study was conducted under special circumstances due to the restrictions imposed during the COVID-19 pandemic. During that period, patients may have been keener to use telemedicine to ensure that they were being followed up on. Therefore, the results obtained may not be generalizable outside of those circumstances.

Another limitation of our study is that our population was relatively young (mean age 44.80, SD 17.49 years) and healthy (mean 6MWD 471.20, SD 125.51 m). The distribution of age and 6MWD was wide but skewed, with 62% (34/55) of the patients being below 50 years of age and 72% of the median 6MWD per patient being above 400 m. Therefore, further studies targeting unhealthier individuals are advised to confirm usefulness in those populations rather than assume similar results to those we obtained.

### Conclusions

This study was motivated by the need to follow up on patients during the restrictions imposed by the COVID-19 pandemic. The use of the Timed Walk app for remote 6MWTs was helpful, as it allowed clinicians to obtain objective measurements of the status of the patients remotely without the need to see patients in person at the clinic. These measurements were at least partially used in approximately half of the medical decisions, showing that the Timed Walk app is useful in clinical practice.

In the future, the usefulness of the app could also be investigated in a study in which patients at greater risk of worsening health are involved. In such a case, it would be important to design a protocol with periodical data review that leverages data automatically sent by the app. A randomized controlled trial would be needed to compare usual care versus app-aided care and assess differences. The study could be designed for any type of patient who is typically administered a 6MWT, such as patients with cardiology (heart failure or pulmonary hypertension), neurology (multiple sclerosis or Parkinson disease), or respiratory diseases (chronic obstructive pulmonary disease).

## Abbreviations

6MWD: 6-minute walk distance
6MWT: 6-minute walk test
CONSORT: Consolidated Standards of Reporting Trials
GNSS: global navigation satellite system
MAU: Malmö University
OUH: Oxford University Hospitals National Health Service Foundation Trust
